# Vaginal Vault Prolapse in an Elderly Woman

**DOI:** 10.7759/cureus.34341

**Published:** 2023-01-29

**Authors:** Nidhi Shah, Anuja Bhalerao

**Affiliations:** 1 Department of Obstetrics and Gynaecology, NKP Salve Institute of Medical Sciences and Research Centre, Nagpur, IND

**Keywords:** sacral colpopexy, vaginal vault prolapse, abdominal sacrocolpopexy, mccall’s culdoplasty, vaginal vault, surgical approach, vault prolapse

## Abstract

Vaginal vault prolapse is a painful condition in which the vaginal cuff descends. This report presents a case of a 65-year-old obese and diabetic female who was suffering from a third-degree vault prolapse. Conventionally used non-surgical treatments, such as exercises for the pelvic floor, are not as effective as surgical approaches for the treatment of third-degree vault prolapse. Post-hysterectomy vaginal vault prolapse can be treated safely and effectively with abdominal sacral colpopexy using a permanent mesh. Due to several risk factors, such as grand parity, advancing age, and poor lifestyle mainly involving exercise to strengthen pelvic floor musculature, the vaginal route of surgery was employed, which was found to be effective, and thus the treatment was successful. In conclusion, such individualized as well as unique approaches to such rare cases can produce efficacious results.

## Introduction

Vaginal vault prolapse is defined by the International Continence Society as the "observation of descent of the vaginal vault (cuff scar after hysterectomy)" [1]. One of the primary factors that pose a risk for vault prolapse is the pre-existing defect in the pelvic floor prior to hysterectomy. Patients with vault prolapse are prone to have coexisting pelvic floor abnormalities such as cystocele, rectocele, and enterocoele. Because of the related bowel, urinary, and sexual problems, prolapse affects the lives of the patients. Hence, it is crucial to carefully evaluate and guide the patient before planning the treatment [2].

Management of vault prolapse can be done either by a non-surgical approach, which is not curative but may help to control symptoms in the patients, or through a surgical approach. In the non-surgical approach that includes minimizing heavy lifting, pelvic floor muscle training by a physiotherapist, and vaginal pessaries, prolapse is reduced by eliminating factors such as constipation and chronic cough. On the other hand, the surgical option includes the correction of vault prolapse via either the vaginal or the abdominal route [3]. Based on the parity, patient's age, comorbidities, history of surgery, amount of physical activity, and sexual activity, the route of surgery should be chosen.

## Case presentation

A 65-year-old post-menopausal woman came with complaints of something coming out of her vagina for six years. The patient described a medical history of diabetes mellitus for nine years and recently is on medication along with a surgical history of vaginal hysterectomy for uterine prolapse eight years ago in addition to a tubal ligation procedure done previously. The patient had five parity and underwent home deliveries in a squatting position. This patient was at risk due to several factors such as increasing age, lifestyle, and grand parity.

**Figure 1:**
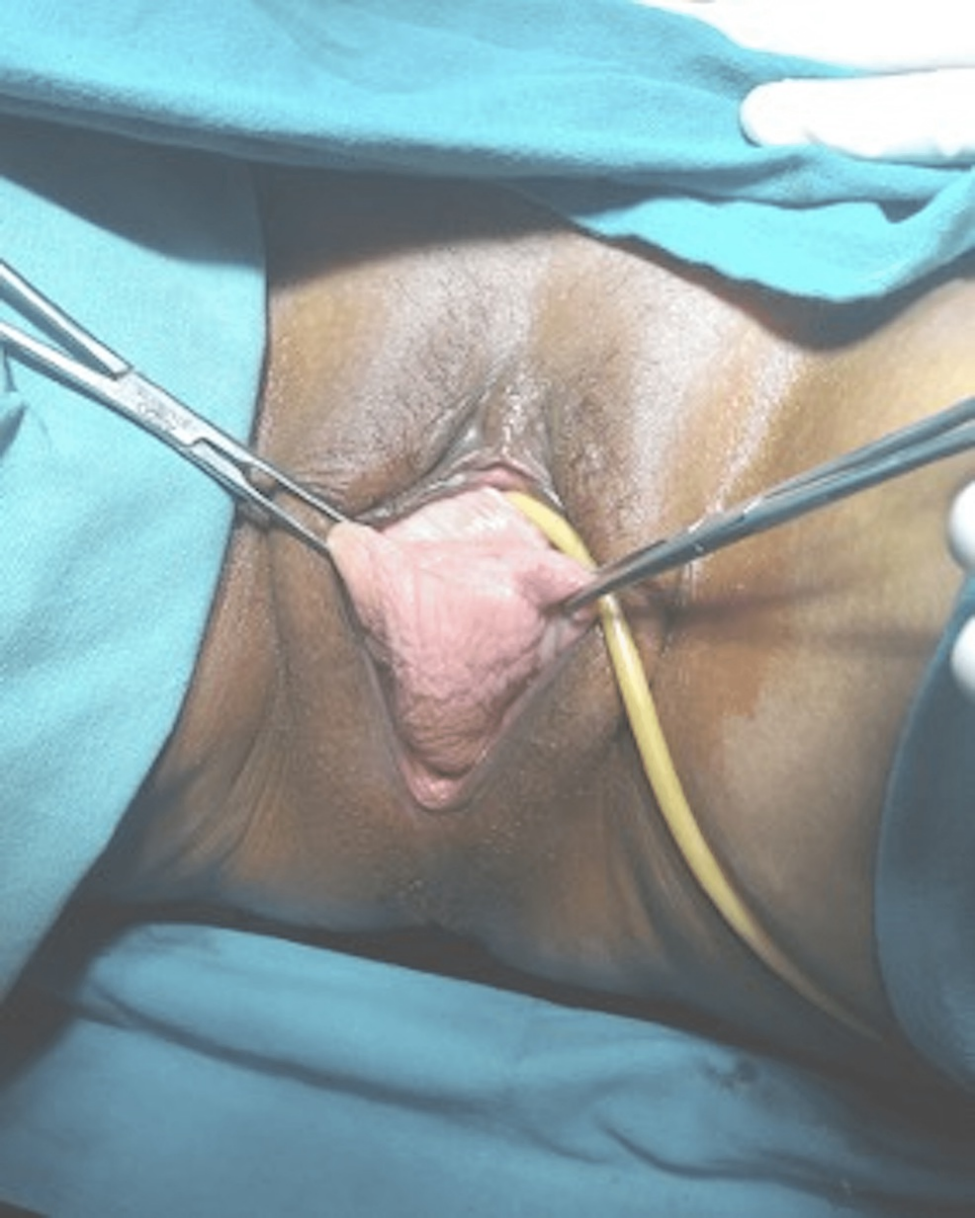
Third-degree vaginal vault prolapse according to the Pelvic Organ Prolapse Quantification.

On examination, all the vital parameters were within normal limits. The patient was obese with a BMI of 31 kg/m^2^. Moreover, on local examination, third-degree vault prolapse was found without any discharge or erosion (Figure 1). The extent of prolapse, i.e., the lowest point of the prolapse extending past the hymen but not the total vaginal length, was assessed by employing a standardized system known as the Pelvic Organ Prolapse Quantification (POP-Q), which also aids in evaluating outcomes of surgical as well as non-surgical treatment and it is also useful for clinical research purposes [2].

The patient underwent abdominal sacrocolpopexy in which the vagina was packed, the abdomen was opened, and anterior dissection up to the levator ani was made, which followed posterior dissection along with the opening of the peritoneum. This was followed by mesh Y-shaped proline repair attaching the anterior longitudinal ligament of the sacrum. The patient adhered well to the surgical intervention and the patient responded well to the treatment during follow-up visits.

## Discussion

In the case of vaginal vault prolapse, previous pelvic floor repairs, grand parity, and age have been found to be the major risk factors that were all present in this woman’s case. Patients come with symptoms of discomfort and urinary symptoms, which can also affect their day-to-day activity.

Non-surgical management of vaginal vault prolapse conventionally includes pelvic floor exercises as well as shelf and ring pessaries. However, there is not enough evidence suggesting the role and impact of pelvic floor exercises in improving the patient's condition [2,4]. Non-surgical management may not be entirely curative but may help in improving the quality of life for patients suffering from vault prolapse; hence, it is opted by many women who want to avoid surgical alternatives.

Surgical management can be done either via the vaginal route or the abdominal route. The vaginal approach includes techniques like McCall’s culdoplasty in which the pouch of Douglas is obliterated by suspending the vault into the uterosacral ligaments' sources [3]. It also includes sacrospinous fixation as well as Iliococcygeal fixation in which the vaginal apex is fixed to iliococcygeal fascia below the ischial spine [5]. Other techniques such as uterosacral suspension, in which the vaginal cuff is fixed to the uterosacral ligaments with sutures positioned at the level of the ischial spines, and infracoccygeal sling sacropexy, which is a less encroaching procedure that does not cause much discomfort to the patient, may be used for the treatment of vault prolapse [6].

The abdominal approach includes techniques like abdominal sacrocolpopexy in which a synthetic, autologous, or allograft prosthesis is placed retroperitoneally between the sacral promontory and vaginal vault. This produces exceptional outcomes as it results in the restoration of normal capacity as well as the axis of the vagina in patients with vaginal vault prolapse [7]. Laparoscopic sacrocolpopexy is another technique that provides surgical details by offering good exposure and thus preventing blood loss, hence it is an excellent modality to perform surgery on the pelvic floor [8]. Severe vaginal vault prolapse can be corrected by using a technique named colpocleisis in which the vaginal canal is closed, thus preventing the prolapse [9].

Non-surgical methods of correction have not been found to be as effective as surgical methods but these methods are given a trial in elderly frail patients who may not be able to tolerate major surgery [2,4]. Post-hysterectomy vaginal vault prolapse can be treated safely and effectively with abdominal sacral colpopexy using a permanent mesh. The available literature on outcomes of abdominal surgery suggests that this treatment approach has the lowest recurrence rate but has been found to be associated with high rates of mortality and increased risks of complications [10-12]. Abdominal sacrocolpopexy may have a longer duration of hospital stay, but it maintains the vaginal axis, which is not the case with the vaginal approach [7].

In this case, due to several factors, such as grand parity, advancing age, and poor lifestyle, the vaginal route of surgery was employed. The vaginal approach has a higher incidence of incontinence post-operatively and a higher rate of prolonged catheterization. Laparoscopy is superior as it has faster recovery, lesser blood loss, and shorter hospital stays, but it has a longer learning curve [8]. Mesh erosion is a common complication in the follow-up period. But nowadays, with the use of polypropylene, this incidence has reduced. Therefore, there is no general agreement on the management and treatment approaches for curing vault prolapse, but there is a consensus on the need to assess and evaluate individual cases and the peculiar circumstances of different patients, as it has been found to produce effective results [2].

## Conclusions

Therefore, to summarize, abdominal sacrocolpopexy is safe and effective for the management of vaginal vault prolapse post-hysterectomy, preferably using a polypropylene mesh. In this case, due to the complex background of the patient as well as various risk factors, abdominal sacrocolpopexy was successful in treating third-degree vaginal vault prolapse and was found to be an efficacious treatment alternative. In conclusion, such individualized and unique approaches to such peculiar cases can produce efficacious results.
